# Patients’ obesity is linked to prolonged thyroidectomy operative time

**DOI:** 10.1007/s00423-025-03789-3

**Published:** 2025-07-02

**Authors:** Ben Eliachar, Ohad Ronen

**Affiliations:** 1 Azrieli Faculty of Medicine, Bar-Ilan University, Safed, Israel; 2 Department of Otolaryngology Head and Neck Surgery, Galilee Medical Center, POB 21, Nahariya, 2210001 Israel

**Keywords:** Thyroid gland, Thyroidectomy, Operative time, Body mass index (BMI), Obesity

## Abstract

**Purpose:**

Obesity is a prevalent condition with potential implications for surgical outcomes, including thyroidectomy. This study investigated the relationship between body mass index (BMI) and the duration of thyroidectomy surgery and length of hospital stay.

**Method:**

A retrospective analysis of patient records. Data included patient age, sex, procedure type, surgery duration, length of hospital stay, histological results, and BMI. Statistical analysis methods (t-tests, ANOVA, Spearman/Pearson correlation, linear regression) were used. Patients were categorized into groups based on BMI. The primary outcome was the relationship between BMI and surgical outcomes. The research was conducted in a tertiary care academic medical center over 7 years (2016–2022). Included were patients who underwent a thyroidectomy performed by otolaryngology residents (*N* = 232).

**Results:**

Surgery duration was significantly prolonged in obese patients, with an average increase of 1.3 min per BMI category (p-value < 0.01), resulting in a mean difference of approximately 10 min between normal weight and obese patients. Length of hospital stay was significantly extended for overweight and obese patients compared to the non-obese group (p-value < 0.01), with an average extension of 0.9 days between normal weight and obese groups.

**Conclusions:**

Obesity is associated with prolonged thyroidectomy surgery duration and length of hospital stay. Our findings highlight the importance of considering BMI in surgical planning and resource allocation. These results can inform preoperative counseling and perioperative management of obese patients undergoing thyroidectomy. Further research is needed to investigate specific factors contributing to prolonged surgery in obese patients.

## Introduction

The global rise in obesity necessitates consideration of its impact on surgical outcomes [1]. In many Western countries, over half the population is overweight or obese [2–4]. This poses significant healthcare challenges, with obesity serving as a risk factor for numerous diseases and impacting surgical practices [3]. Thyroidectomy, a common procedure performed for various thyroid pathologies, is no exception.

Thyroidectomy is frequently performed on obese patients, though the procedure poses unique challenges due to excess adipose tissue [5]. Surgical difficulties arise from obscured anatomical landmarks, particularly the recurrent laryngeal nerve (RLN) and parathyroid glands, potentially increasing risks of nerve injury and postoperative hypocalcemia [6–8]. Despite these concerns, recent studies have yielded inconsistent findings regarding the association between obesity and complications following thyroidectomy, with some demonstrating no significant impact on RLN injury or hypocalcemia [9–12]. Similarly, the relationship between obesity and thyroidectomy duration remains unclear, with conflicting evidence across studies [9–13]. 

Given the inconclusive literature, this study aimed to investigate the relationship between BMI and both thyroidectomy duration and length of hospital stay. We hypothesized that higher BMI will be associated with:

- Prolonged surgery duration: due to challenges in accessing anatomical structures and potential for increased bleeding.
- Extended hospital stay: due to potential for delayed wound healing and increased drainage volume in obese patients.

Understanding these associations can inform surgical planning, resource allocation, and patient counseling for optimized healthcare delivery in the context of a growing obese population.

## Materials and methods

### Study description

This retrospective cohort study was conducted at a tertiary care medical center, investigating electronic medical records of patients who underwent thyroidectomy between 2016 and 2022.

### Participants

All patients aged 18 years and above who underwent primary thyroidectomy (excluding cervical lymph node dissection) during the defined period were eligible for the study. Exclusion criteria included presence of previous neck surgery, additional surgical procedures during thyroidectomy (e.g., central lymph node dissection), incomplete medical records.

### Data collection

Data were extracted from electronic medical records, including: demographics (age, sex), preoperative BMI calculated from admission weight and height, surgery duration (time from skin incision to closure), length of hospital stay (defined from the end of surgery to discharge), final histological diagnosis from pathology reports, and occurrence of postoperative complications including RLN injury and clinically significant hypocalcemia. For statistical purposes, Non-Invasive Follicular Thyroid Neoplasm with Papillary-like nuclear features (NIFTP) was categorized as benign.

### BMI categorization

Based on WHO definitions, BMI was categorized as follows: Group A: Normal/Underweight (BMI < 25 kg/m²), Group B: Overweight (BMI 25–30 kg/m²), Group C: Obese (BMI > 30 kg/m²).

### Statistical analysis

One-way ANOVA was used to compare differences in surgery duration and length of stay across BMI groups, adjusting for age, sex, and procedure type as potential confounders. Spearman/Pearson correlation was used to assess the relationship between BMI and each outcome variable (surgery duration, length of stay, thyroid gland size), adjusting for age and sex. Linear regression was used to further model the association between BMI and outcomes, while controlling for potential confounders. This allowed for estimation of the effect size of BMI on surgery duration and length of stay, providing a more nuanced understanding of the relationship.

### Ethical considerations

The study was approved by the Institutional Review Board Galilee Medical Center’s Institutional Review Board (***010623).

## Results

### Patient characteristics

A total of 232 patients underwent thyroidectomy (partial or total) between 2016 and 2022. Nineteen patients were excluded: 15 underwent cervical dissection and two had previous neck surgery, leaving 215 for analysis, see Table 1.

**Table 1:** Patient demographics and clinical features, categorized by BMI groups

	Group A BMI < 25, *n* = 59	Group B 25 < BMI < 30, *n* = 72	Group C BMI > 30, *n* = 84
Gender			
Male (*n* = 52)	11	22	19
Female (*n* = 163)	48	50	65
Age groups			
18–65	51	57	61
65–80	8	15	23
Time			
Operation time (minutes)	119.9	117.9	129.4
Hospital stay (days)	3.3	4.0	4.2
Surgery			
Partial thyroidectomy (*n* = 173)	44	57	72
Complete thyroidectomy (*n* = 42)	15	15	12
Postoperative complications			
Transient RLN injury	1.7%	4.2%	3.6%
Transient Hypocalcemia	0%	2.8%	0%

### Surgery duration

**Fig. 1:**
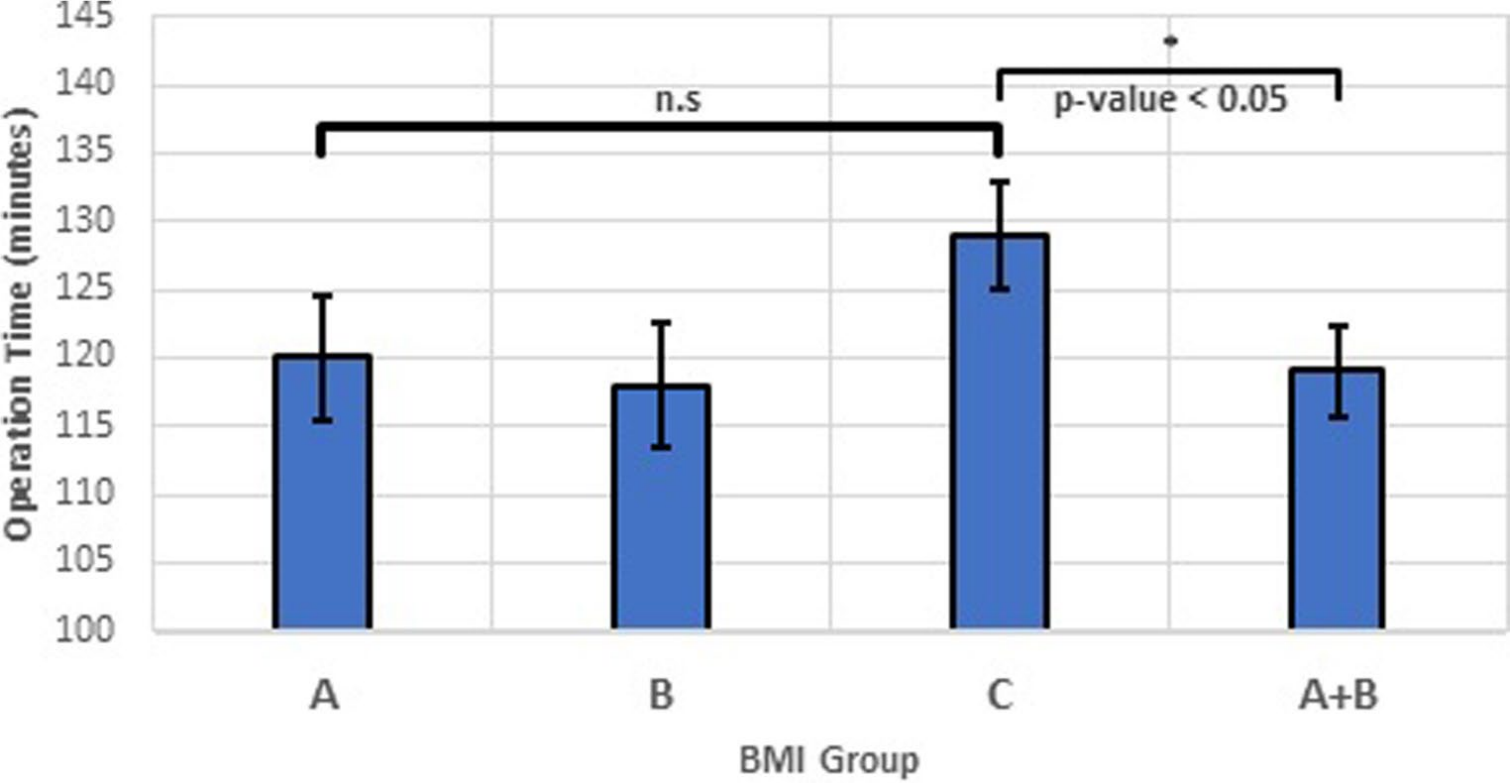
Comparison of Surgery Duration across BMI Categories. Bars represent the mean and standard deviation of surgery duration for each BMI category (Group A: normal/underweight, Group B: overweight, Group C: obese). Error bars depict the standard deviation. One-way ANOVA found no significant difference between all three groups (*p* = 0.118). Combining categories A and B (“Combined: normal/underweight & overweight”) and comparing against group C (“Obese”) revealed a significant difference in surgery duration (*p* = 0.039)

One-way ANOVA comparing BMI categories did not reveal a statistically significant difference in surgery duration (*p* = 0.118). However, when merging groups A and B (normal/underweight and overweight) and comparing against obese (group C), a significant difference in surgery times was observed (*p* = 0.039), see Fig. 1. The mean difference in operative time between normal weight (Group A) and obese patients (Group C) was approximately 10 min (119.9 vs. 129.4 min), representing an 8% increase.

### Type of surgery

To address whether the type of thyroidectomy (partial versus total) was distributed differently across patient BMIs, we compared the mean BMI between these two procedure groups. Of the 215 patients included in the final analysis, 173 underwent partial thyroidectomy and 42 underwent total thyroidectomy, see Table 1. A One-Way ANOVA revealed no statistically significant difference in mean BMI between patients undergoing partial thyroidectomy (Mean BMI = 29.06 ± 5.32) and those undergoing total thyroidectomy (Mean BMI = 28.17 ± 6.51, *p* = 0.414). This suggests that the type of procedure performed was not significantly associated with patient BMI in this cohort.

### Histological diagnosis

Final histological diagnoses were categorized as benign or malignant. Of the patients with available definitive histology, 73.7% were classified as benign (including 4 cases of NIFTP) and 26.3% were malignant. A One-Way ANOVA was performed to determine if there was a difference in BMI based on pathology. No statistically significant difference in mean BMI was found between patients with benign pathology (mean BMI = 29.9 ± 5.71) and those with malignant pathology (mean BMI = 28.8 ± 5.18; *p* = 0.344). This suggests that patient BMI was not significantly different between those with benign and malignant thyroid conditions in this study group.

### Postoperative complications

Postoperative complications, including RLN injury and clinically significant hypocalcemia, were reviewed. Transient RLN injury was identified in 3.25% of patients. A One-Way ANOVA revealed no statistically significant difference in mean BMI between patients who experienced a RLN injury (mean BMI = 30.0 ± 7.04) and those who did not (mean BMI = 28.8 ± 5.53, *p* = 0.674). Clinically significant transient postoperative hypocalcemia occurred in 0.93% of patients. Similarly, there was no statistically significant difference in mean BMI between patients who developed transient hypocalcemia (mean BMI = 27.4 ± 0.990) and those who did not (mean BMI = 28.9 ± 5.597, *p* = 0.227).

### Hospitalization duration

**Fig. 2:**
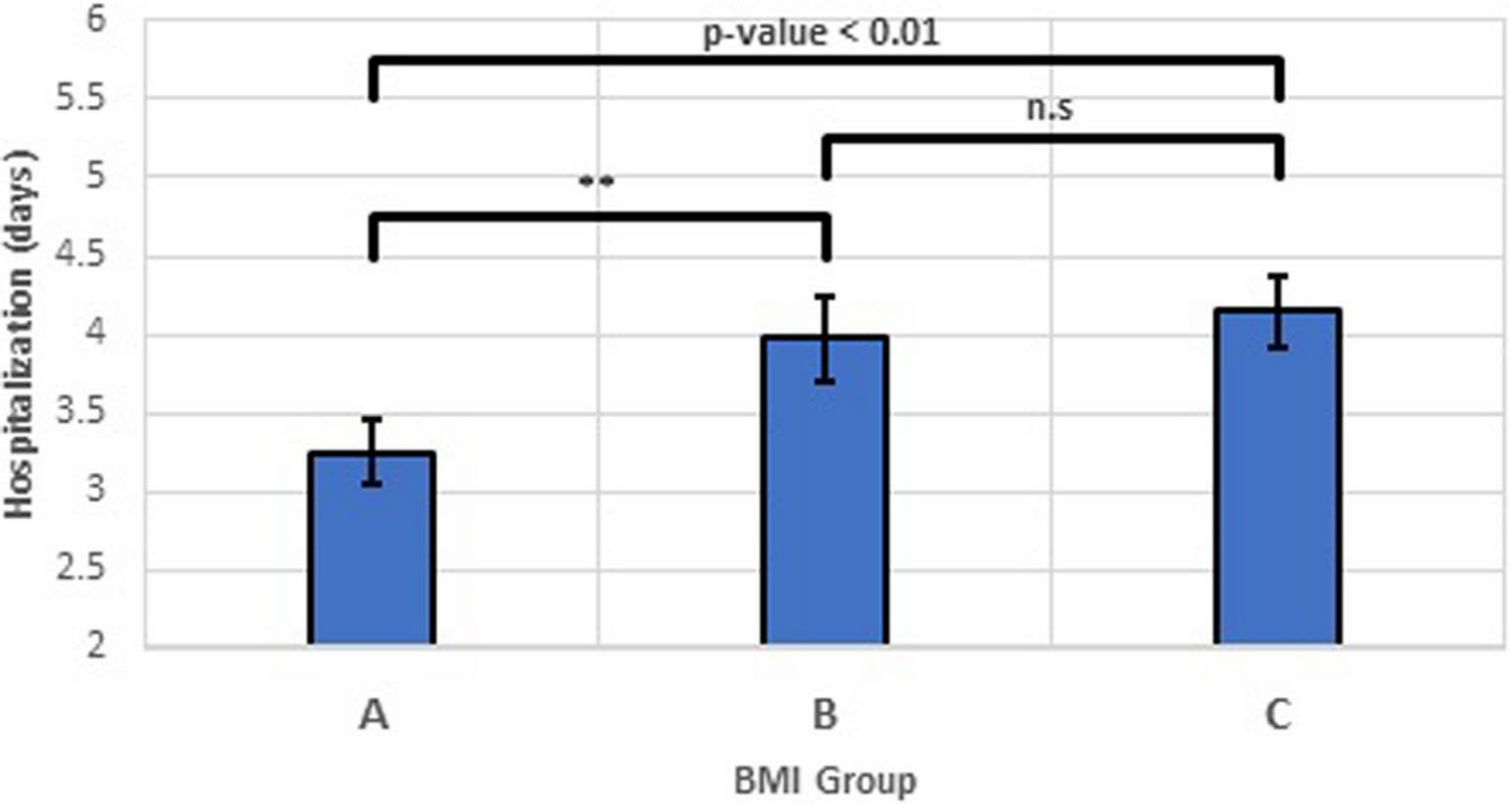
Hospitalization Duration by BMI Category after Surgery. Bars represent the mean and standard deviation of hospitalization duration for each BMI category (Group A: normal/underweight, Group B: overweight, Group C: obese). Error bars depict the standard deviation. One-way ANOVA revealed a statistically significant difference in hospitalization duration across all three BMI groups (*p* = 0.009), with a mean difference of 0.9 days (27% increase) between normal weight and obese patients

One-way ANOVA showed a statistically significant difference in hospitalization duration after surgery across BMI categories (*p* = 0.009), see Fig. 2. The mean hospitalization was 3.3 days for Group A, 4.0 days for Group B, and 4.2 days for Group C, representing a 0.9-day (27%) increase between normal weight and obese patients.

### Correlations

A positive, statistically significant correlation was identified between surgery duration and length of hospitalization (Pearson’s *r* = 0.282, *p* < 0.001), see Fig. 5 in the appendix.

A weak, non-significant correlation was found between BMI as a continuous variable and surgery duration (Pearson’s *r* = 0.118, *p* = 0.083), see Fig. 6 in the appendix.

No significant difference in BMI was observed between genders (*p* = 0.396), however, male patients had significantly longer surgery times compared to females (*p* = 0.008), see Figs. 7 and 8, in the appendix, respectively.

**Fig. 3:**
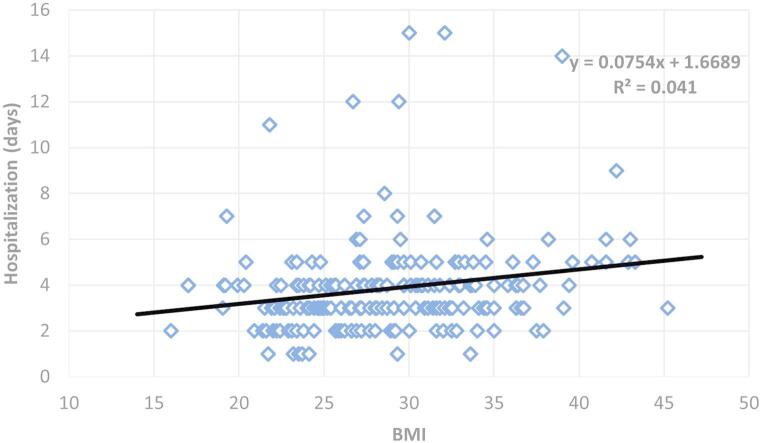
Relationship between BMI and hospitalization time. The scatter plots display individual data points representing subject BMI (x-axis) and hospitalization duration (y-axis). The trendline shows a significant negative correlation (Pearson’s *r* = 0.203). Statistical analysis revealed a positive correlation between subject BMI and hospitalization duration, indicating that patients with higher BMI tend to have longer hospital stays (*p* = 0.003)

BMI as a continuous variable positively correlated with hospitalization duration (Pearson’s *r* = 0.203, *p* = 0.003), see Fig. 3.

**Fig. 4:**
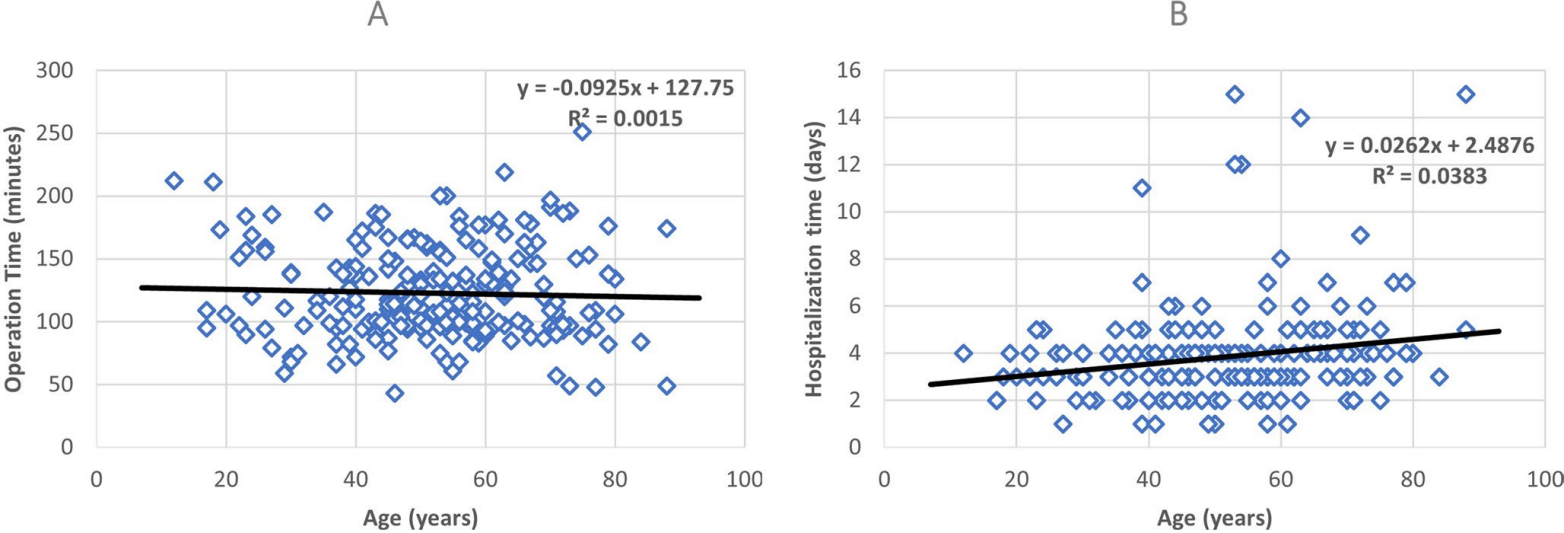
Relationship between age and surgery and hospitalization time. The scatter plots display individual data points representing subject age (x-axis) and surgery/hospitalization duration (y-axis). **A**. The trendline shows a nonsignificant negative correlation (Pearson’s *r* = −0.039). Statistical analysis revealed no significant correlation between subject age and surgery duration (*p* = 0.573). **B**. The trendline shows a significant positive correlation (Pearson’s *r* = 0.196). Statistical analysis revealed a positive correlation between subject age and hospitalization duration, indicating that patients with older age tend to have longer hospital stays (*p* = 0.004)

**Fig. 5:**
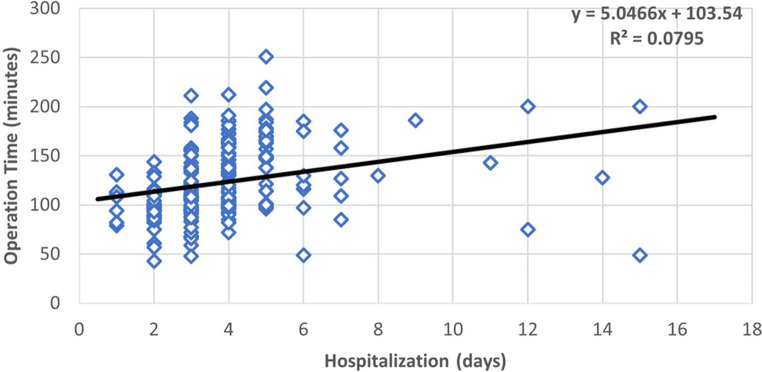
Relationship between surgery and hospitalization time. The scatter plots display individual data points representing hospitalization duration in days (x-axis) and surgery duration in minutes (y-axis). We found a positive, statistically significant correlation between surgery duration and length of hospitalization (Pearson’s *r* = 0.282, *p*< 0.001)

**Fig. 6:**
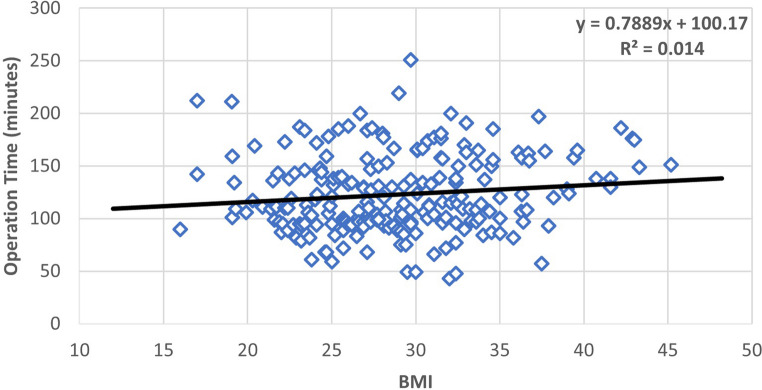
Relationship between surgery time and BMI. The scatter plots display individual data points representing BMI (x-axis) and surgery duration in minutes (y-axis). We found a weak, non-significant correlation between BMI as a continuous variable and surgery duration (Pearson’s *r* = 0.118, *p* = 0.083)

**Fig. 7:**
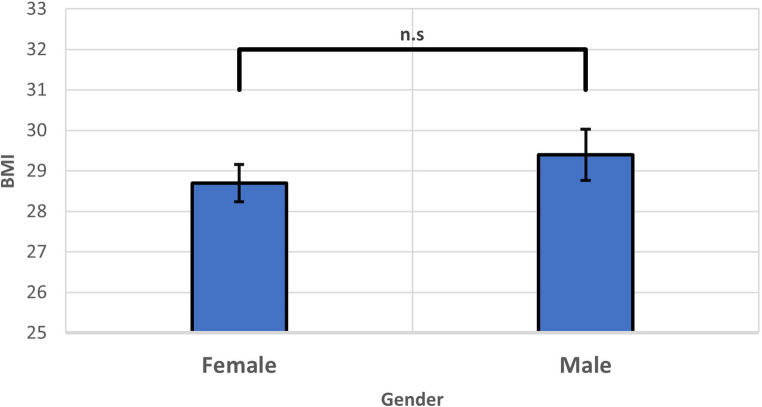
BMI differences between genders. No significant difference in BMI was observed between genders (*p* = 0.396)

**Fig. 8:**
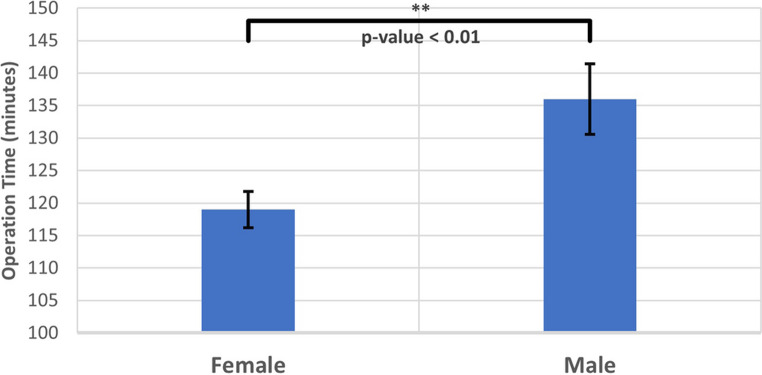
Surgery time differences between genders. Male patients had significantly longer surgery times compared to females (*p* = 0.008)

Subject age showed no significant correlation with surgery duration (Pearson’s *r* = −0.039, *p* = 0.573), but did show a positive correlation with hospitalization duration (Pearson’s *r* = 0.196, *p* = 0.004), see Fig. 4A and B, respectively.

#### Linear regression

Two separate linear regression models were conducted:

##### Hospitalization duration

Age and BMI were both significant predictors (*p* = 0.020 and 0.024, respectively), partially explaining hospitalization duration, see Table 2 in the appendix.

##### Surgery duration

Age and gender were identified as significant predictors (*p* = 0.009 and 0.002, respectively), partially explaining surgery time, see Table 3 in the appendix.

## Discussion

This study investigated the association between BMI and thyroidectomy outcomes in an adult population. While previous studies reported mixed findings regarding the effect of obesity on surgery duration and hospitalization, this research aimed to address limitations of existing literature by using a recent, larger sample [9–11, 14, 15]. 

Our findings revealed a non-significant difference in surgery duration across all BMI categories (Fig. 1), potentially due to advancements in surgical techniques and technologies like hemostasis tools. However, when comparing obese patients (group C) to the remaining population (groups A + B), a significant increase in surgery time was observed (Fig. 1). This difference of approximately 10 min represents an 8% increase in operative time, which may have implications for surgical scheduling and resource allocation in high-volume thyroid surgery centers. It suggests that the impact of obesity becomes prominent only at higher BMI levels, aligning with our hypothesis. This finding contributes to the understanding of surgical complexities in patients with obesity. Factors such as challenging anatomical exposure of important anatomical structures, e.g. the RLN and parathyroid glands, due to excess adipose tissue may contribute to this increase. Furthermore, the type of thyroidectomy performed (partial vs. total) was not found to be associated with patient BMI, mitigating concerns those results would be limited to that a disproportionate number of more extensive procedures in any particular BMI group might confound the primary outcome of operative time related to BMI (Table 1). The surgeons may also find it more difficult to operate on patients with obese habitus, stand further away from the operative bed, struggling to visualize the thyroid and tissues located deeper in the neck, patients’ shoulders and arms extending over the surgery table, and possible shorter necks.

The overall rates of key postoperative complications in our cohort were as follows: transient RLN injury occurred in 3.25% of patients, and clinically significant transient hypocalcemia was observed in 0.93%. Importantly, our analysis did not find an association between higher BMI and an increased risk of either transient RLN injury or transient postoperative hypocalcemia. However, the relatively small number of patients experiencing these complications, particularly hypocalcemia, limits the statistical power to detect subtle differences if they exist and warrants cautious interpretation.

Hospital stay was significantly longer for overweight and obese patients compared to normal/underweight individuals (Figs. 2 and 3). A 0.9-day (27%) increase between normal weight and obese patients represents a clinically meaningful difference that impacts healthcare costs, resource utilization, and patient experience. This finding supports our hypothesis and aligns with prior evidence suggesting increased risk of postoperative complications in obese patients, potentially leading to delays in discharge. One significant factor contributing to prolonged hospital stays could be related to postoperative drainage. Obese patients, particularly those with larger goiters, may experience increased serous fluid accumulation post-surgery, leading to higher or more prolonged drain output. Clinical decisions to keep drains in place until output is minimal would directly contribute to an extended hospital stay. While our current study did not specifically quantify drain output against BMI and length of stay, this remains a plausible mechanism and an important area for future investigation. Other factors such as higher rates of surgical site infections or delayed wound healing, also reported in literature concerning obese surgical patients, may contribute as well.

Interestingly, age emerged as a significant predictor of hospitalization duration but not surgery time (Fig. 4A and B). While older patients exhibited longer hospital stays, no correlation was found with surgery duration. This suggests that age may act as a confounding variable in studies examining the relationship between BMI and surgery duration, highlighting the importance of controlling for relevant factors in future research.

### Limitations and future directions

Our study acknowledges certain limitations. Although based on a larger sample compared to some prior studies, further research incorporating data from multiple hospitals and considering additional factors like medical history, surgical technique, and thyroid gland size, as well as specific postoperative parameters like drain output volume and duration, could provide more comprehensive insights into the complex interplay between BMI and thyroidectomy outcomes. While this study offers insights into the impact of BMI on operative time and length of stay, it lacks sufficient data to assess its association with RLN injury or hypocalcemia. Given the low incidence of these complications in our cohort, studies with larger sample sizes would be beneficial in defining these correlations with greater statistical power [5, 10, 11, 16]. 

## Conclusion

This study highlights the potential impact of obesity on thyroidectomy outcomes. While surgery duration may not be significantly affected across all BMI categories, obese patients experience longer hospital stays. These findings emphasize the importance of considering BMI when planning surgery and preparing patients for potential prolonged hospitalization. The observed differences in operative time (10 min) and hospital stay (0.9 days) between normal weight and obese patients have meaningful implications for resource allocation, surgical scheduling, and patient counseling. Future research exploring the influence of additional factors and implementing multi-center designs can further refine our understanding of the relationship between obesity and thyroidectomy outcomes.

## Data Availability

Data is available upon a reasonable request from the corresponding author.

## Appendix

**Table 2:** Linear regression for predicting hospitalization time

Model Fit Measures						
Model	R	R^2^				
1	0.253	0.0643				
Model Coefficients - Hospitalization (days)						
			95% Confidence Interval			
Predictor	Estimate	SE	Lower	Upper	*t*	*P*
Intercept	0.8513	0.87132	–0.86628	2.5689	0.977	0.330
BMI	0.0604	0.02564	0.00981	0.1109	2.354	0.020
Age	0.0209	0.00921	0.00272	0.0390	2.267	0.024
Genders	0.1353	0.32211	–0.49968	0.7702	0.420	0.675

**Table 3:** Linear regression for predicting operation time

Model Fit Measures						
Model	R	R^2^				
1	0.279	0.0780				
Model Coefficients - Operation Time (minutes)						
			95% Confidence Interval			
Predictor	Estimate	SE	Lower	Upper	*t*	*p*
Intercept	64.3583	16.746	31.339	97.377	3.843	< .001
BMI	1.2618	0.482	0.312	2.211	2.620	0.009
Age	–0.0285	0.170	–0.364	0.307	–0.167	0.867
Genders	17.8051	5.670	6.625	28.985	3.140	0.002
